# Pilot Study to Assess the Ability of a 4-Week, Home-Based, Electrical Muscle Stimulation Program to Improve Lower Extremity Function and Reduce Sarcopenia in Older Individuals With Cancer

**DOI:** 10.1016/j.arrct.2025.100479

**Published:** 2025-06-01

**Authors:** Hisashi Kosaka, Tome Ikezoe, Kimitaka Hase, Yutaka Kimura, Takumi Miyauchi, Tung Thanh Lai, Khanh Van Nguyen, Kyoko Inoue, Moriyasu Takada, Hideyuki Matsushima, Gozo Kiguchi, Hidekazu Yamamoto, Kosuke Matsui, Megumi Taketani, Tomoyuki Shirai, Masaki Kaibori

**Affiliations:** a Department of Hepatobiliary Surgery, Kansai Medical University, Hirakata.; b Faculty of Rehabilitation, Kansai Medical University, Hirakata.; c Department of Physical Medicine and Rehabilitation, Kansai Medical University, Hirakata.; d Health Science Center, Kansai Medical University Hospital, Hirakata.; e MTG Co, Ltd, Nagoya, Japan.

**Keywords:** Electric stimulation, Lower extremity, Rehabilitation, Sarcopenia

## Abstract

**Objective:**

To assess the effectiveness of home-based self-rehabilitation using a portable electrical muscle stimulation (EMS) device for improving severe sarcopenia by improving lower extremity function.

**Design:**

The effect of 4 weeks of EMS training on improving lower extremity function was compared between patients divided into 2 groups based on baseline lower extremity function. Self-rehabilitation was carried out with a portable EMS device, the SIXPAD Foot Fit, and each session lasted 15-23 minutes. Lower extremity function was assessed with the Short Physical Performance Battery (SPPB).

**Setting:**

University hospital.

**Participants:**

The study included 50 older outpatients (N=50) with a mean age of 75 years; 98% had hepatobiliary cancer, and 38% were men.

**Interventions:**

Not applicable.

**Main Outcome Measures:**

Patients were divided into 2 groups based on baseline SPPB value (SPPB ≤ 9 or SPPB >9). Lower extremity function was observed prospectively during 4 weeks of home-based self-rehabilitation.

**Results:**

EMS was used consistently, with a median duration of use of 28 days. In the baseline SPPB>9 group, lower extremity function was not significantly improved (SPPB, 11.0-12.0; *P*=.290). In contrast, significant improvement was observed in the baseline SPPB ≤ 9 group, and the total score (SPPB score, 8.0-9.0; *P*=.001) and 2 of its 3 components, balance (balance score, 3.0-4.0; *P*=.009) and gait speed (gait speed score, 3.0-4.0; *P*=.002), improved significantly. Sit-to-stand ability did not improve (Sit-to-stand score, 1.0-2.0; *P*=.060). As a result, the proportion of patients with severe sarcopenia was initially 66.7% but decreased significantly to 36.4% (*P*=.002).

**Conclusions:**

A home-based, self-rehabilitation program using a portable EMS device may improve lower extremity function and attenuate sarcopenic status in older patients with cancer with reduced lower extremity function.

Patients with cancer are often in a state of sarcopenia, where muscle mass is reduced, and the decline in quality of life caused by this loss of muscle mass has a negative effect on survival time.^1^ As functional impairment adversely affects most patients with cancer, rehabilitation care can have a significant effect on patients’ functioning and quality of life, thus necessitating rehabilitation interventions concomitant with cancer treatment.^2^ However, there are several issues with continuing rehabilitation during anticancer therapy, with treatment-related adverse events, lack of time, and fatigue reported to be the main barriers to exercise for patients with a variety of cancers.^3^ Therefore, it is important for patients with cancer to establish a home-based self-rehabilitation method that they can continue safely, easily, and effectively at any time.

Electrical muscle stimulation (EMS) is considered safe and limits or reverses the sarcopenic process and its structural changes by modulating the molecular processes involved in the progression of muscle atrophy.^4^ The effects of EMS seem to vary depending on the range of electrical stimulation of the muscles. Reports on nonportable EMS capable of providing a wide range of exercise through electrical stimulation have shown that its implementation in hospitalized patients improves lower extremity strength and function.^5^^,^^6^ In contrast, a report on portable EMS, which can provide a smaller range of exercise at home, showed that at home self-rehabilitation using this on the quadriceps muscles of patients who have undergone liver transplant was effective in maintaining quadriceps muscle thickness after surgery, but did not affect lower limb muscle strength or function.^7^ To carry out effective home-based self-rehabilitation for older patients with sarcopenia who are unable to participate frequently in exercise rehabilitation programs and actively continue exercise training, EMS equipment that is portable and capable of electrically stimulating a wide range of muscles is required.

This study aimed to investigate the feasibility and/or potential effectiveness of a portable EMS device capable of stimulating a wide range of the lower extremities in home-based self-rehabilitation for improving severe sarcopenia through the improvement of lower extremity function.

## Methods

### Patients

This was a prospective observational study to examine the effectiveness of 4 weeks of home-based self-rehabilitation using electric muscle stimulation to improve lower extremity function in older patients with cancer. This study was approved by the institutional review board of Kansai Medical University (IRB approval number: 2021322). It was performed in accordance with the Declaration of Helsinki. Fifty-three patients with cancer visiting Kansai Medical University Hospital between March 2023 and July 2024 were enrolled in this study. Patients were recruited during outpatient visits for cancer treatment and participated in the study after providing consent. Three of the 53 patients were excluded because their cancer condition worsened, and they discontinued home-based self-rehabilitation within 2 weeks from use. The remaining 50 patients were divided into 2 groups based on lower extremity function as defined by baseline Short Physical Performance Battery (SPPB) value (baseline SPPB ≤9, declined function; baseline SPPB>9, healthy lower extremity function). Lower extremity function in these 2 groups was measured prospectively over time during 4 weeks of home-based self-rehabilitation. This study was approved by the institutional review board of Kansai Medical University (Approval number: 2021322).

### Measures of physical performance

Short Physical Performance Battery is a 3-part performance-based test (gait speed, chair stand, balance) to assess lower extremity function: standing balance in positions of side-by-side stance, semi-tandem stance, and full tandem stance; walking speed on a 4-meter course regardless of the use of a walking stick; and standing up from a chair 5 times without using the arms.^8^^,^^9^ Each test is scored on a scale of 0-4, with a maximum score of 12 indicating better lower extremity function. SPPB was measured at baseline, at 2 weeks, and finally at 4 weeks to evaluate lower extremity function.

### Measures of muscle strength and muscle mass

Knee extension, toe grip strength, and hand grip strength (HGS) were measured with dynamometers at baseline and 4 weeks. The bioelectrical impedance analysis device assessed lean body mass of the trunk and both legs, skeletal muscle mass, skeletal mass index (SMI), and phase angle at baseline and 4 weeks.

### Sarcopenic status

The Asian Working Group for Sarcopenia (AWGS) 2019 consensus defined sarcopenia as age-related loss of muscle mass, plus low muscle strength, and/or low physical performance.^10^ After the AWGS 2019 sarcopenia diagnosis, we defined sarcopenia as both low muscle mass (SMI<7.0 kg/m^2^ in men and <5.7 kg/m^2^ in women) and either low muscle strength (HGS<28.0 kg in men and <18.0 kg in women) or low physical performance (SPPB ≤9). Patients who met all 3 of these criteria were defined as having severe sarcopenia.^11^

### EMS

SIXPAD FootFit 2 and Foot Fit 3, portable EMS devices,^a^ were used for 4 weeks of home-based self-rehabilitation. These EMS devices are widely used, both in Japan and overseas, and can be easily operated while seated, even by older patients with cancer, by simply placing their bare feet on the device and operating the remote control, allowing them to exercise both lower extremities safely while remaining seated for 15-23 minutes. These EMS devices were programmed to deliver a group of 5 square-wave pulses at a frequency of 20 Hz, with each pulse having a width of 100 μs. Patients operated the EMS equipment themselves at home and recorded device usage. Patients adjusted the stimulation intensity themselves according to the tolerable muscle pain during EMS (range of stimulation level: 1-25).

### Statistical analysis

Data are expressed as numbers with percentages or medians with interquartile ranges (IQRs). The Shapiro–Wilk test was used to assess the normality of continuous variables. The Student *t* test was performed after the Levene test for normally distributed data, and the Mann–Whitney *U* test was performed for nonnormally distributed data. The Kruskal–Wallis test was used to assess the significance of differences between groups. The Fisher exact test was used for nominal scale data. Comparisons were considered statistically significant at *P*<.05. All statistical analyses were performed with the IBM SPSS^b^ version 22 software package for Windows.

## Results

### Background characteristics

The background characteristics of the entire cohort, the baseline SPPB ≤9 group, and the baseline SPPB>9 group are shown in table 1. In the overall cohort, the majority were older patients, with a median age of 75 years. The rates of biliary tract cancers and hepatocellular carcinoma were 86.0% and 12.0%, respectively; 88.0% of patients received drug therapy. The median body mass index was 21.0 kg/m^2^. At baseline, 70.0% of patients were diagnosed with sarcopenia, and 44.0% had severe sarcopenia. Concerning determinants of sarcopenia by the AWGS 2019 guideline, the median SMI, HGS, and SPPB were lower than the standard value (SMI for men, 6.2 kg/m^2^; women, 5.4 kg/m^2^; HGS for men, 20.0 kg; women, 11.0 kg; SPPB, 8.0). EMS was used consistently, with a median number of days of use over the 4-week study period of 28.0 (24.3-28.0) days, with a mean stimulation level of 16.9, and EMS-related adverse events were not observed. The 2 subgroups did not differ significantly, except on the prevalence of severe sarcopenia, which was significantly higher in the baseline SPPB ≤9 group than in the baseline SPPB>9 group (severe sarcopenia: 66.7% vs 0.0%, respectively; *P*=.001).

**Table 1:** Background characteristics overall and by baseline SPPB score.

Parameter	All	Baseline SPPB		*P* Value
		SPPB ≤9	SPPB >9	
Number of patients	50	33	17	–
Age	75.0 (69.0-79.0)	75.0 (68.5-79.5)	74.0 (69.5-78.5)	.659
Gender, male	19 (38.0)	14 (42.4)	5 (29.4)	.369
Body mass index	21.0 (18.8-23.1)	21.0 (18.9-23.5)	21.0 (18.1-23.5)	.467
Primary disease				.538
Biliary tract cancer	43 (86.0)	29 (87.9)	14 (82.4)	
Hepatocellular carcinoma	6 (12.0)	3 (9.1)	3 (17.6)	
Gastric cancer	1 (2.0)	1 (3.0)	0 (0.0)	
Current treatment				.339
Chemotherapy	44 (88.0)	28 (84.8)	16 (94.1)	
Periodic follow-up	6 (12.0)	5 (15.2)	1 (5.9)	
Baseline sarcopenic status				.001
Nonsarcopenia	15 (30.0)	11 (33.3)	4 (23.5)	
Sarcopenia	13 (26.0)	0 (0.0)	13 (76.5)	
Severe sarcopenia	22 (44.0)	22 (66.7)	0 (0.0)	
Determinants of sarcopenia				
Skeletal muscle index, kg/m^2^				
male	6.2 (5.6-6.9)	6.2 (5.4-7.0)	6.2 (5.9-6.9)	.849
female	5.4 (5.0-5.8)	5.6 (5.0-5.9)	5.2 (4.9-5.8)	.491
Hand grip strength, kg				
male, right	19.5 (17.4-25.0)	19.5 (16.5-25.3)	24.0 (16.8-24.8)	.095
left	19.8 (14.6-22.1)	18.2 (14.3-22.3)	20.5 (16.0-22.5)	.143
female, right	10.5 (9.0-13.1)	10.3 (8.8-11.3)	12.3 (9.1-14.6)	.415
left	11.5 (10.0-14.0)	11.0 (9.8-12.3)	13.0 (11.5-15.5)	.415
SPPB	8.0 (7.0-11.0)	8.0 (6.5-8.0)	11.0 (11.0-12.0)	.001
EMS usage status				
Number of times used (d)	28.0 (24.3-28.0)	28.0 (23.0-28.0)	28.0 (25.5-28.0)	.397
Stimulation level, range: 1-25	16.9 (14.2-20.3)	16.7 (14.7-20.8)	17.1 (13.1-18.6)	.463
Adverse event	0 (0.0)	0 (0.0)	0 (0.0)	–

NOTE. Data are displayed as number with percent or median with interquartile range. The Mann–Whitney *U* test and the Fisher exact test were used to assess the significance of differences between groups.

### Improvement in lower extremity function using EMS for 4 weeks of home-based self-rehabilitation

**Fig 1:**
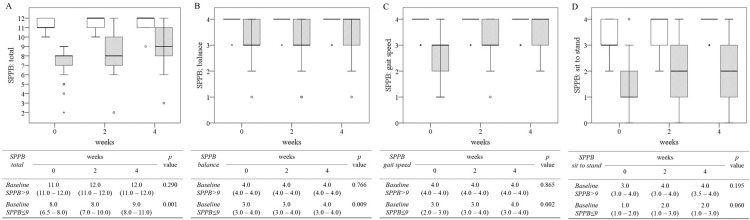
Changes in the short physical performance battery score and its 3 components over time through 4 weeks of home-based self-rehabilitation using EMS. Total SPPB score (A) and scores of its 3 components: balance (B), gait speed (C), and sit-to-stand (D), according to baseline SPPB score>9 (white box, n=17) or ≤9 (dotted box, n=33). Values are displayed as median with interquartile range. The Kruskal–Wallis test was used to assess the significance of differences between groups.

The changes in SPPB and its 3 components over 4 weeks of home-based self-rehabilitation using EMS are displayed in table 2. The median (IQR) SPPB score improved significantly from 8.0 (7.0-11.0) to 11.0 (9.0-11.0) points (*P*=.007). This improvement was attributable to improvements in balance ability and gait speed, but not sit-to-stand ability. The changes in SPPB and its 3 components over time according to baseline SPPB ≤9 or baseline SPPB >9 are shown in figure 1. In the baseline SPPB >9 group, no significant improvement was observed in the total score or its components (SPPB, 11.0-12.0; *P*=.290). In contrast, the improvement effect was remarkable in the baseline SPPB ≤9 group (SPPB, 8.0-9.0; *P*=.001), and the total score and the components of balance ability and gait speed, improved significantly; sit-to-stand ability did not. More detailed measurements for the SPPB ≤9 group are shown in table 3. Compared with baseline values, after 4 weeks, tandem balance improved by 4.3 seconds (5.7-10.0s) and gait speed increased by 0.17 meters/second (0.67-0.84m/s), whereas sit-to-stand changed by 1.1 seconds (16.6-15.5s), which was not significant.

**Table 2:** Changes in the SPPB and its 3 components through 4 weeks of home-based self-rehabilitation using EMS.

Parameter	Wk			*P* Value
	0	2	4	
Number of patients	50	50	50	
SPPB				
Total score, 0-12	8.0 (7.0-11.0)	10.0 (8.0-11.0)	11.0 (9.0-11.0)	.007
Balance score, 0-4	4.0 (3.0-4.0)	4.0 (3.0-4.0)	4.0 (3.8-4.0)	.047
Gait speed score, 1-4	3.0 (2.0-4.0)	3.0 (3.0-4.0)	4.0 (3.0-4.0)	.012
Sit-to-stand score, 1-4	2.0 (1.0-3.0)	3.0 (2.0-3.0)	3.0 (2.0-4.0)	.115

NOTE. Data are displayed as median with interquartile range. The Kruskal–Wallis test was used to assess the significance of differences between groups.

**Table 3:** Detailed changes in the actual measurement values of the 3 components of the SPPB assessment over time through 4 weeks of home-based self-rehabilitation using EMS in patients with baseline SPPB scores ≤9.

Parameter	Wk			*P* Value
	0	2	4	
Number of patients	33	33	33	
Balance				
Parallel, s	10.0 (10.0-10.0)	10.0 (10.0-10.0)	10.0 (10.0-10.0)	>.99
Semi-Tandem, s	10.0 (10.0-10.0)	10.0 (10.0-10.0)	10.0 (10.0-10.0)	>.99
Tandem, s	5.7 (4.2-10.0)	7.5 (5.5-10.0)	10.0 (7.8-10.0)	0.005
Gait speed, m/s	0.67 (0.55-0.81)	0.78 (0.65-0.89)	0.84 (0.67-0.91)	0.004
Sit-to-stand, s	16.6 (14.7-20.4)	15.5 (12.7-16.9)	15.5 (13.0-17.6)	0.117

NOTE. Data are displayed as median with interquartile range. The Kruskal–Wallis test was used to assess the significance of differences between groups.

### Improvement of sarcopenic status using EMS for 4 weeks of home-based self-rehabilitation

**Fig 2:**
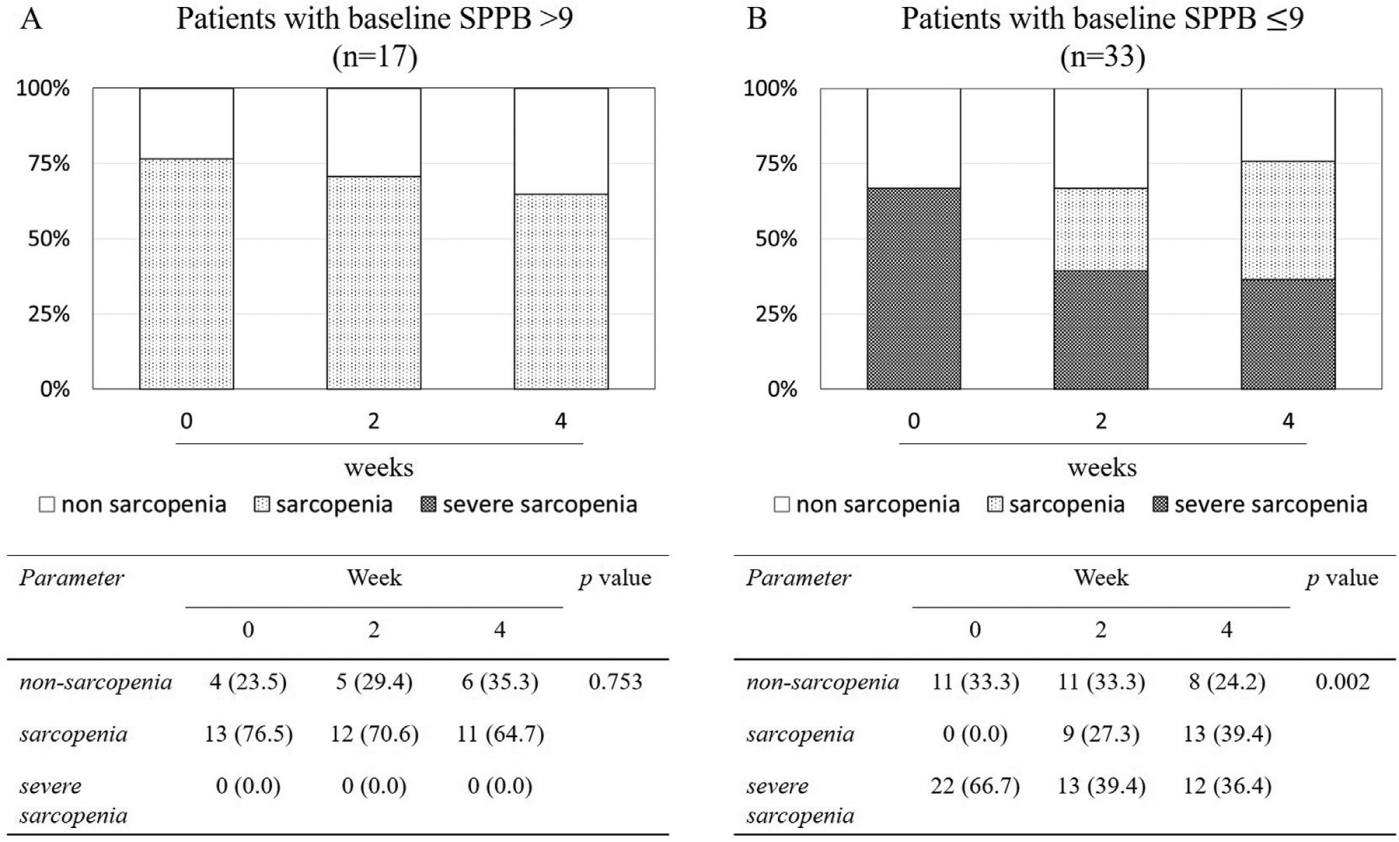
Changes in sarcopenic status over time through 4 weeks of home-based self-rehabilitation using EMS. Sarcopenic status is shown according to baseline SPPB >9 (A) and SPPB ≤9 (B). Values are displayed as number with percentage. The Fisher exact test was used to assess the significance of differences between groups.

Changes in sarcopenic status over time through 4 weeks of home-based self-rehabilitation using EMS by baseline SPPB are displayed in figure 2. In the baseline SPPB >9 group, no patients were initially in a severe sarcopenic state, and the rate of sarcopenia decreased from 76.5% to 64.7% which was not significant (*P*=.753). In contrast, in the SPPB ≤9 group, the proportion of patients with severe sarcopenia was initially 66.7%, but decreased significantly to 36.4% after 4 weeks of home-based self-rehabilitation using EMS (*P*=.002). Table 4 shows the changes in the 3 components for sarcopenia over 4 weeks in the SPPB ≤9 group. Among the components for sarcopenia, no improvement was observed in SMI and HGS; only SPPB improved significantly.

**Table 4:** Changes in the 3 components of the sarcopenia assessment after 4 weeks of home-based self-rehabilitation using EMS in patients with baseline SPPB scores ≤9.

Parameter	Wk		*P* Value
	0	4	
Number of patients	33	33	
Determinants of sarcopenia			
Skeletal muscle mass, kg/m^2^			
male	6.2 (5.4-7.0)	6.6 (5.6-6.9)	.830
female	5.6 (5.0-5.9)	5.2 (4.8-5.8)	.461
Hand grip strength, kg			
male, right	19.5 (16.5-25.3)	18.5 (14.3-21.8)	.375
left	18.2 (14.3-22.3)	16.4 (12.5-21.5)	.616
female, right	10.3 (8.8-11.3)	11.0 (10.0-12.0)	.298
left	11.0 (9.8-12.3)	11.0 (9.5-14.5)	.641
SPPB, range: 1-12	8.0 (6.5-8.0)	9.0 (8.0-11.0)	.001

NOTE. Data are displayed as median with interquartile range. The Mann–Whitney *U* test was performed to assess the significance of differences between groups. Thirty-three patients including 13 men and 20 women.

### Changes in muscle strength and volume

No significant improvements in muscle strength were observed. No significant changes were observed in knee extension and toe grip strength before and after 4 weeks of EMS training (table 5). In addition, no significant improvements in muscle mass were observed. No significant changes were observed in SMI, trunk, or lower limb muscle mass before and after 4 weeks of EMS training.

**Table 5:** Changes in muscle strength and volume after 4 weeks of home-based self-rehabilitation using EMS.

Parameter	Wk		*P* Value
	0	4	
Number of patients	50	50	
Knee extension strength, kg			
male, right	11.8 (8.9-13.7)	11.4 (10.0-12.8)	.822
left	10.9 (8.8-13.7)	11.2 (10.2-14.0)	.480
female, right	8.7 (7.5-10.1)	9.4 (7.9-10.7)	.083
left	8.7 (8.0-10.2)	9.6 (8.5-11.2)	.131
Toe grip strength, kg			
male, right	9.6 (4.0-11.1)	8.2 (5.5-10.6)	.775
left	9.3 (6.9-11.1)	8.3 (6.4-10.6)	.730
female, right	7.5 (5.5-8.4)	7.8 (6.2-8.7)	.309
left	7.9 (6.2-8.5)	7.6 (6.2-8.6)	.885
Lean body mass, trunk, kg			
male	18.6 (16.4-19.5)	18.5 (16.4-19.7)	.938
female	13.8 (12.9-15.1)	13.8 (12.8-14.9)	.988
Lean body mass, leg, kg			
male, right	6.2 (5.8-7.4)	6.5 (6.0-7.5)	.518
left	6.2 (5.7-7.2)	6.5 (5.9-7.2)	.641
female, right	4.7 (4.4-5.2)	4.8 (4.4-5.2)	.914
left	4.7 (4.4-5.3)	4.8 (4.4-5.5)	.686
Phase angle,°			
male	3.4 (3.1-3.7)	3.1 (2.8-3.8)	.578
female	3.4 (2.8-3.6)	3.3 (2.7-3.8)	.965
Skeletal muscle mass, kg			
male	22.3 (19.7-24.1)	22.7 (19.2-24.1)	.940
female	17.2 (16.5-18.2)	17.4 (16.6-17.9)	.757

NOTE. Data are displayed as median with interquartile range. The Mann–Whitney *U* test was performed to test the significance of differences between groups. Fifty patients, including 19 men and 31 women.

In addition, there were no significant improvements in immune nutritional status and liver function, such as albumin–bilirubin score, neutrophil-to-lymphocyte ratio, prognostic nutritional index, platelet-to-lymphocyte ratio, C-reactive protein-to-albumin ratio, and C-reactive protein–albumin–lymphocyte index (supplemental table S1, available online only at http://www.archives-pmr.org/).

## Discussion

This study demonstrated that 4 weeks of home-based self-rehabilitation using SIXPAD FootFit, a portable EMS device, improved lower extremity function and attenuated sarcopenic status in older patients with cancer. Improvement was most notable in patients with lower extremity function whose baseline SPPB was 9 or less.

It is known that a decline in lower extremity function significantly impacts the survival of older patients with cancer.^2^^,^^12^ Rehabilitation to maintain and improve lower extremity function is necessary, but it is often difficult for patients with cancer to carry out rehabilitation. Anticancer therapy presents several challenges for patients with cancer to adhere to rehabilitation; treatment-related adverse events, time constraints, and fatigue have been identified as the primary obstacles to exercise for patients with various cancers.^3^ Adherence to rehabilitation among patients with cancer can be unsatisfactory, especially unsupervised home-based rehabilitation compared with supervised center-based rehabilitation.^13^ The mean adherence to unsupervised exercise programs among patients with cancer was reported to be 75%-76%.^14^^,^^15^ In contrast, in this study, the median number of days of unsupervised home-based self-rehabilitation using EMS over 4 weeks was 28, which was satisfactory. The reason for this high adherence rate is presumably because even older patients with cancer can easily perform exercise through electrical stimulation at home at their convenience with portable EMS. Because effective rehabilitation can be continued without the supervision of a physical therapist, it may be expected that this will have a ripple effect, such as reducing the rising medical costs required for rehabilitation for the elderly and resolving the manpower shortage of physical therapists. Additionally, this research may provide the basis for new supportive care that can be continued throughout the treatment of older patients with cancer undergoing chemotherapy.

In recent years, there have been increasing numbers of research reports on the positive rehabilitative effects of EMS.^16^ The therapeutic effects of EMS have been reported to vary depending on the range of muscles stimulated with EMS. Whereas nonportable EMS can stimulate a wide area effectively and improve lower extremity strength and function, conventional portable EMS can only stimulate a small area, limiting its effectiveness.^5^, ^6^, ^7^^,^^17^ In contrast, the portable EMS unit used in this study was lightweight (1.38 kg), yet it was still effective in improving lower extremity function. Among the factors evaluated by the SPPB, no improvement was observed in the sit-to-stand test, but favorable effects were observed in gait speed and tandem balance. These results suggest that the EMS used in this study delivered electrical stimulation through the sole and therefore had little effect on the quadriceps, which affects the sit-to-stand test, but may have affected the triceps surae, ankle strength, and plantar flexor stability, which are closer to the sole. Previous reports have shown that ankle strength and plantar flexor stability are important for forward and backward balance ability,^18^ and we speculate that the improvement in tandem balance in this study was also likely because of improved ankle strength and plantar flexor stability. In addition, we speculate that the improvement in walking speed is because of improved forward/backward balance ability and improved push-off force while walking because of improved plantar flexor stability.

Although lower extremity function improved, there was no significant increase in lean body mass of the legs, knee extension, and toe grip strength after 4 weeks of EMS training. Two randomized controlled trials suggest that nutritional supplementation may be one factor that improves muscle strength and mass. In hospitalized older patients with sarcopenia, in addition to rehabilitation, a whey protein-based nutritional formula enriched with leucine and vitamin D has been reported to increase walking speed and muscle mass.^19^ Another report showed that combining EMS training with a whey-based nutritional supplement (whey protein, polyphenols, and omega-3 fatty acids) improved gait speed and knee extension strength in older patients with sarcopenia.^20^ These reports suggest that adding supplements to home-based self-rehabilitation using a portable EMS device may result in more effective rehabilitation, but this must be confirmed in future randomized controlled trials.

Although much remains unknown regarding the signaling pathways by which electrical stimulation by EMS affects muscles, there have been several reports about key signaling factors. Myokines are characterized as cytokines and other peptides produced, expressed, and released by muscle fibers that exert paracrine or endocrine effects.^21^ Myostatin, a myokine, is known to have a negative effect on regulating skeletal muscle growth and is suppressed by electrical stimulation of muscles using EMS.^17^^,^^22^ Myonectin, a myokine, improved skeletal muscle function by activating increased mitochondrial function by electrical stimulation of muscles using EMS in a rat model.^23^

No adverse events were observed in this study, and the degree of muscle stimulation was self-regulating, so there were no reports of pain or discomfort. No serious adverse events related to EMS have been reported in the literature.^5^^,^^16^ A tingling sensation was reported but was not clinically significant, and one other superficial burn occurred because of an improper EMS setting.^24^ There are currently no reports that EMS itself has a negative effect on the progression of cancer.

### Study limitations

This was a single-arm, nonrandomized, nonblinded trial evaluating the effectiveness of home-based self-rehabilitation using portable EMS at a single institution. In addition, only a few types of cancer were considered, and the sample was not only small, but also one of convenience. Although the small sample size limited our ability to apply multiple comparisons correction, it is the large number of statistical tests that increases the risk of an inflated type I family-wise error rate. Further high-quality randomized controlled trials are needed to draw stronger conclusions.

## Conclusions

Four weeks of home-based self-rehabilitation using a portable EMS device may improve lower extremity function and attenuate sarcopenic status in older patients with cancer. This study may provide a basis for establishing an alternative rehabilitation option.

## Suppliers

a. SIXPAD Foot Fit; MTG Co, Ltd. b. SPSS, version 22.0; IBM.

## Disclosure

The investigators have no financial or nonfinancial disclosures to make in relation to this project.

## Appendix Supplementary materials

Image, application 1

## Footnotes

Supplementary material associated with this article can be found, in the online version, at doi:10.1016/j.arrct.2025.100479.
