# Supplementary material for: Pilot Study to Assess the Ability of a 4-Week, Home-Based, Electrical Muscle Stimulation Program to Improve Lower Extremity Function and Reduce Sarcopenia in Older Individuals With Cancer
Source: Arch Rehabil Res Clin Transl. 2025 Jun 1;7(3):100479. doi: 10.1016/j.arrct.2025.100479 (PMC12447214; doi:10.1016/j.arrct.2025.100479)
Supplement: Supplementary file 1 [file mmc1.docx]

| Supplemental table 1. Changes in blood tests following 4 weeks of home-based self-rehabilitation using electrical muscle stimulation | | | |
| --- | --- | --- | --- |
| *Parameter* | Weeks | | *p* value |
|  | 0 | 4 |  |
| *WBC,×10^2^μL* | 44.0 (35.0 – 62.3) | 50.5 (35.8 – 64.8) | 0.442 |
| *Hb, g/dL* | 10.2 (9.0 – 11.4) | 10.1 (9.0 – 11.6) | 0.912 |
| *PLT,×10^4^μL* | 16.7 (10.9 – 23.3) | 16.9 (13.5 – 20.1) | 0.788 |
| *Albumin, g/dL* | 3.6 (3.1 – 3.9) | 3.7 (3.3 – 3.9) | 0.769 |
| *Total bilirubin, mg/dL* | 0.6 (0.4 – 0.8) | 0.5 (0.4 – 0.8) | 0.749 |
| *AST, U/L* | 25.0 (19.0 – 33.5) | 23.0 (18.8 – 31.3) | 0.508 |
| *ALT, U/L* | 17.5 (13.0 – 25.3) | 16.0 (12.0 – 19.3) | 0.123 |
| *Creatine kinase, U/L* | 0.79 (0.61 – 1.01) | 0.84 (0.57 – 1.03) | 0.772 |
| *CRP, mg/dL* | 0.33 (0.08 – 1.00) | 0.60 (0.17 – 1.55) | 0.424 |
| *ALBI score* | -2.51 (-2.73 – -1.92) | -2.45 (-2.74 – -2.05) | 0.809 |
| *NLR* | 2.2 (1.3 – 2.9) | 2.4 (1.4 – 3.5) | 0.324 |
| *PNI* | 42.9 (37.2 – 46.4) | 41.7 (37.8 – 46.4) | 0.584 |
| *PLR* | 113.7 (84.0 – 178.9) | 141.7 (92.7 – 183.8) | 0.331 |
| *CAR* | 0.09 (0.02 – 0.34) | 0.15 (0.04 – 0.44) | 0.476 |
| *CALLY* | 1.11 (0.39 – 9.16) | 0.69 (0.22 – 3.40) | 0.270 |
| Data are displayed as median with interquartile range. The Mann-Whitney U test was performed to test the significance of differences between groups.  IQR, Interquartile range; WBC, White blood cell; Hb, Hemoglobin; PLT, Platelet count; AST, aspartate transaminase; ALT, alanine transaminase; CRP, C-reactive protein; ALBI score, albumin–bilirubin score; NLR, Neutrophil-to-lymphocyte ratio; PNI, prognostic nutritional index; PLR, platelet-to-lymphocyte ratio; CAR, C-reactive protein-to-albumin ratio; CALLY index, CRP–albumin–lymphocyte index. *Three patients could not be evaluated because of their condition. | | | |
